# Systems Biology Reveals Relevant Gaps in Fc-γR Expression, Impaired Regulatory Cytokine Microenvironment Interfaced With Anti-*Trypanosoma cruzi* IgG Reactivity in Cardiac Chagas Disease Patients

**DOI:** 10.3389/fmicb.2018.01608

**Published:** 2018-07-30

**Authors:** Juliana de A. S. Gomes, Fernanda F. de Araújo, Daniele M. Vitelli-Avelar, Renato Sathler-Avelar, Paula S. Lage, Ana P. B. Wendling, Isabela N. P. C. do Vale, João C. P. Dias, Silvana M. Elói-Santos, Andréa Teixeira-Carvalho, Olindo A. Martins-Filho

**Affiliations:** ^1^Grupo Integrado de Pesquisas em Biomarcadores, Instituto René Rachou, Fundação Oswaldo Cruz, FIOCRUZ, Belo Horizonte, Brazil; ^2^Laboratório de Biologia das Interações Celulares, Departamento de Morfologia, Instituto de Ciências Biológicas, Universidade Federal de Minas Gerais, Belo Horizonte, Brazil; ^3^Programa de Pós-graduação em Sanidade e Produção Animal nos Trópicos, Universidade de Uberaba, Uberaba, Brazil; ^4^Triatomíneos, Instituto René Rachou, Fundação Oswaldo Cruz, FIOCRUZ, Belo Horizonte, Brazil; ^5^Departamento de Propedêutica Complementar, Faculdade de Medicina, Universidade Federal de Minas Gerais, Belo Horizonte, Brazil

**Keywords:** Chagas disease, Fc receptors, *Trypanosoma cruzi*, cytokines, immune response

## Abstract

The systems biology approach has become an innovative tool when it comes to shedding light on the complex immune response underlying the development/maintenance of distinct clinical forms of Chagas disease. The goal of this study was to describe an integrative overview of Fc-γR expression, cytokine microenvironment and anti-*Trypanosoma cruzi* IgG interface in indeterminate-(IND) and cardiac-(CARD) patients. Data demonstrated that IND displayed an overall higher Fcγ-R expression (CD16; CD32; CD64) on neutrophils-(NEU), along with (CD16; CD64) on monocytes-(MON) as compared to CARD. Additionally, CARD presented an increased expression of CD32 in B-cells. While preserved frequency of IL-10-producing cells was observed in IND, decreased levels of IL-10^+^ phagocytes and enhanced TNF^+^ MON and NK-cells were observed in CARD. *T. cruzi*-antigen recall *in vitro* induces a general decrease of Fc-γR expression in Chagas disease patients, especially in CARD. Moreover, *T. cruzi*-antigen stimuli triggered a concomitant increase of IFN-γ^+^NEU/TNF^+^NK-cells and IL-10^+^MON/IL-10^+^B-cells in IND. Biomarker signatures further emphasized the contrasting Fc-γR expression and cytokine microenvironment observed in Chagas disease patients with distinct clinical forms. Up-regulation of Fc-γR expression (CD16 on NEU;MON;NK) was observed in IND, whereas a general decrease was reported for CARD. Moreover, while a mixed cytokine microenvironment (TNF; IL-10) was observed in IND, CARD presented a contrasting profile with up-regulation of TNF^+^NEU and IL-12^+^NEU. Integrative network analysis revealed a distinct assemblage of biomarkers, with CARD presenting a large number of negative internode connectivity in comparison with IND. The relevant gaps in Fc-γR expression and impaired regulatory cytokine microenvironment interfaced with the anti-*T. cruzi* IgG reactivity throughout an exacerbated negative connectivity may account for the development/maintenance of the clinical status of cardiac Chagas disease.

## Introduction

Chagas disease, caused by *Trypanosoma cruzi*, remains a serious public health problem and affects about 10 million people in Latin America (World Health Organization [WHO], 2017). The *T. cruzi* infection induces a broad repertoire of antibodies against both parasite antigens (Kierszenbaum, 1980; Brodskyn et al., 1988; Heath et al., 1990; Kumar and Tarleton, 1998) and self-molecules (Minoprio et al., 1989; Cunha-Neto et al., 1995; Santos-Lima et al., 2001; Lu et al., 2008). Distinct levels of anti-*T. cruzi* antibodies have been observed in patients with different clinical forms of Chagas disease (Cordeiro et al., 2001). However, the precise role that antibodies may play in interconnecting the cellular immune response is not fully understood.

The Fc-γR represent the major interface between humoral and cellular immune responses. These molecules have been involved in activating several functions including phagocytosis, degranulation, cytokines production, and antibodies-dependent cellular cytotoxicity (Ravetch and Kinet, 1991). Therefore, the Fc-γR are essential to the immune system through a link between antibodies and effector functions (Pricop et al., 1997). Three different classes of Fc-γR have been described in humans. The Fc-γRI (CD64), Fc-γRII (CD32), and Fc-γRIII (CD16) are expressed in different immune cells, with distinct Fc-γR isotypes (Qiu et al., 1990). Fc receptors can simultaneously trigger activation and inhibition pathway sets thresholds for cell activation and generate an effective immune response (Ravetch and Lanier, 2000). Our group has previously shown that monocytes from IND presented a putative lower expression of Fc-γR upon *in vitro* culture either in the absence or presence of live trypomastigotes organisms. The down-regulation of Fc-γR expression by monocytes from IND was associated with a lower phagocytic capacity, but not with the anti-*T. cruzi* IgG observed in IND (Gomes et al., 2012). No previous report has addressed the integrative network assembled by distinct patterns of Fc-γR expression or the impact of soluble *T. cruzi* antigens on Fc-γR expression by circulating leucocytes subsets in patients with distinct clinical forms of Chagas disease.

In this study, we investigated the contribution of Fc-γR receptors to the development/maintenance in the chronic phase of Chagas disease. An integrative overview of Fc-γR expression in neutrophils, monocytes, NK cells, B and T lymphocytes was evaluated for IND and CARD patients, as well as their cytokine expression and anti-*T. cruzi* IgG interface. Our data showed that gaps in the Fc-γR expression and the impaired regulatory cytokine microenvironment interfaced with the anti-*T. cruzi* IgG reactivity throughout an exacerbated negative connectivity may account for the development/maintenance of the clinical status of cardiac Chagas disease.

## Materials and Methods

### Study Population

This study was approved by the Ethical Committee at IRR/FIOCRUZ, Belo Horizonte, Minas Gerais, Brazil (CAAE 11-2004). All patients had read and signed the informed consent form, according to Resolution 466/2012 from the Brazilian National Health Council for scientific research with humans. All participants were residents of the municipality of Bambuí, state of Minas Gerais, Brazil. Laboratory and clinical examinations were carried out for diagnosis and to differentiate the clinical forms of late chronic Chagas disease. Conventional serology was performed to establish the positive and negative status of Chagas disease. Standard commercially available serological tests, including indirect hemagglutination and indirect immunofluorescence assays were used as recommended by the Brazilian National Consensus for Chagas disease diagnosis. A total of 32 volunteers (11 males and 21 females, ages ranging from 20 to 70 years) were enrolled in the present investigation, comprising 21 Chagas disease patients and 11 healthy non-infected individuals (NI, 03 males and 08 females) as a control group. According to their clinical records, the Chagas disease patients were categorized into two different groups, referred to as Indeterminate (IND, 03 males and 05 females) and Cardiac (CARD, 05 males and 08 females) clinical forms. Indeterminate Chagas disease patients presented asymptomatic chronic infection with no clinical manifestations other than the positive serological status. Patients included in the CARD group displayed cardiac dysfunction, characteristic of dilated cardiomyopathy defined by a detailed clinical examination, including chest X-ray, electrocardiography, and 24 h Holter monitoring.

### Short-Term Whole Blood Culture *in Vitro*

Heparinized peripheral blood samples (10 mL) were collected from each participant into Vacutainer tubes (BD Pharmingen, San Diego, CA, United States). Short-term whole blood cultures *in vitro* were carried out as previously described by Vitelli-Avelar et al. (2008). Briefly, aliquots of heparinized whole blood samples were incubated in 14 mL polypropylene tubes (Falcon^®^, BD Pharmingen, San Jose, CA, United States) in two distinct conditions, including the control culture-CC, referred as “*Ex vivo,”* and upon stimulation with *T. cruzi*-Ag, referred as “*T. cruzi* antigen recall.” In the *Ex vivo* condition, 500 μL of whole blood were incubated with 500 μL of RPMI-1640 (GIBCO, Grand Island, NY, United States) and 10 μL of Brefeldin A (Sigma, St. Louis, MO, United States) to reach a final concentration of 10 μg/mL. For *T. cruzi* antigen recall *in vitro*, 500 μL of whole blood were pre-incubated with 400 μL of RPMI-1640 and 100 μL of trypomastigote *T. cruzi* soluble antigen (CL strain), prepared as described by Gomes et al. (2003) at a final concentration of 20 μg/mL, for 1 h at 37°C in 5% CO_2_ humidified incubator. Following antigen priming *in vitro*, 10 μL of Brefeldin A were added to each whole blood sample and cultures were incubated for 4 h at 37°C in a 5% CO_2_ humidified incubator.

### Intracytoplasmic Cytokine Analysis by Flow Cytometry

Following short-term whole blood culture *in vitro*, each sample was incubated for 15 min with 200 μL of EDTA (Sigma, St. Louis, MO, United States) to reach a final concentration of 2 mM. The samples were then washed once with 3 mL of FACS buffer (phosphate buffered saline supplemented with 0.5% of bovine serum albumin and 0.1% sodium azide, Sigma, St. Louis, MO, United States). Samples were resuspended with 1 mL of FACS buffer and 400 μL aliquots stained in the dark for 30 min at room temperature with FITC (BD Pharmingen, San Diego, CA, United States) or TC (Caltag, Burlingame, CA, United States) labeled mAbs [anti-CD3 (UCHT1); CD19 (4G7); CD14 (M5E2); CD16 (3G8); CD32 (3D3), and CD64 (10.1)]. After lysing/fix procedure, membrane-stained cells were permeabilized for 30 min at room temperature with FACS perm-buffer (FACS buffer supplemented with 0.5% of saponin). Next, samples were distributed in U-bottom microplates containing 20 μL of PE-labeled anti-cytokine mAbs [anti-TNF-α (MAb11); IL-12p40/p70 (C11.5); IFN-γ (4S.B3); IL-4 (BVD4-1D11); IL-10 (JES3-19F1), and IL-13 (JES10-5A2), BD Pharmingen, San Diego, CA, United States]. After intracytoplasmic cytokine staining, the cells were washed once with FACS perm-buffer, once with FACS buffer, and then fixed with FACS FIX Solution (10 g/L of paraformaldehyde, 10.2 g/L of sodium cacodylate and 6.63 g/L of sodium chloride, pH 7.2, Sigma, St. Louis, MO, United States). Samples were stored at 4°C up to 24 h prior to flow cytometry acquisition. A total of 30,000 events/sample were run in a four-color FACScalibur^TM^ flow cytometer (Becton Dickinson, San Jose, CA, United States) using CellQuest^TM^ software (Franklin Lakes, NJ, United States).

Distinct gating strategies were applied to select leukocyte subsets, including neutrophils, monocytes, NK, and B-cells, as previously described by Vitelli-Avelar et al. (2008). Briefly, neutrophils and monocytes were identified as SSC^high^CD16^high+^ and CD14^high+^ events, respectively. NK and B-cells were identified first by selection of FSC^low^SSC^low^ events followed by the selection of CD5^-^CD19^-^ and CD19^+^ events, respectively. Upon selecting each leucocytes subset, the expression of Fcγ-R as well as the frequency of cytokine-positive was determined. CellQuest^TM^ software was employed for flow cytometry analysis. *Ex vivo* data were reported as mean fluorescence intensity (MFI) for Fcγ-R expression and as percentage of cytokine-positive cells within a given leukocyte subset. Data regarding the impact of *T. cruzi* antigen recall *in vitro* were reported as *T. cruzi* Ag/CC Index for both Fcγ-R expression and cytokine-positive cells.

### Immunofluorescence for Anti-live Trypomastigote IgG by Flow Cytometry

The immunofluorescence assay was carried out as previously described (Cordeiro et al., 2001). Briefly, 5 × 10^5^ parasites were incubated in the presence of serial serum dilutions (1:128 to 1:16,384) at 37°C for 30 min. After incubation, the parasites were washed twice with phosphate buffered saline supplemented with 10% fetal bovine serum and reincubated in the presence of fluorescein isothiocyanate (FITC)-conjugated anti-human IgG antibody at 37°C for 30 min in the dark. After two wash procedures, the FITC-labeled parasites were fixed with a FACS FIX Solution before analysis in the cytometer. The results were expressed as percentage of fluorescent positive parasites (PPFP) along the titration curve. Additionally, the reactivity pattern was further addressed by calculating the proportion of subjects with PPFP >20%, referred to as the positivity ratio.

### Data Mining and Statistical Analysis

Comparative analysis among study groups was performed by Kruskal–Wallis variance analysis followed by Duns’ post-test. The Chi-square test was applied to define differences in proportions between groups. Gray-shaded diagrams were built to calculate the frequency of subjects with high biomarker indices above the global median estimated for the study population. Relevant attributes were identified in biomarker signatures, considering the 50^th^ percentile as the cut-off as proposed by Luiza-Silva et al. (2011). The Spearman correlation test was applied to yield statistic matrix (*p* and *r* values) to assemble the biomarker networks. GraphPad Prism software, version 5.03 (San Diego, CA, United States), was used for statistical analysis and graphic arts. In all cases, significance was considered at *p* < 0.05. Microsoft Excel was used to assemble gray-shaded diagrams. Cytoscape software, version 3.2.0, was used to construct nodal biomarker network layouts for each clinical group. Connecting edges were used to display the association between attributes and identify correlation scores, classified as positive (solid line) or negative (equal dashed line).

## Results

### Differential Expression of Fc-γR by Phagocytes, NK-Cells, and B-Lymphocytes From IND and CARD

**FIGURE 1:**
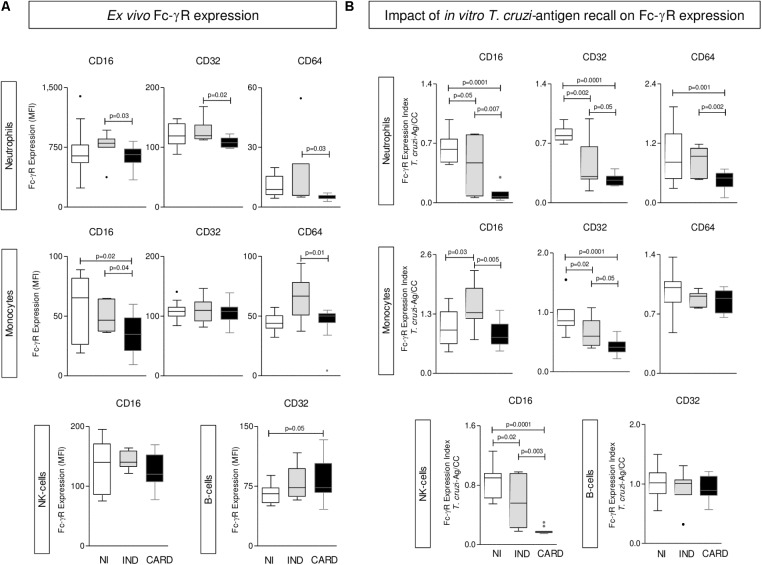
Expression of Fc-γR by phagocytes, NK-cells, and B-lymphocytes. The analysis of Fc-γR expression by neutrophils, monocytes, NK-cells, and B-cells were performed in whole blood samples collected from indeterminate (IND, 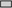 = 8) and cardiac (CARD, 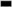 = 13) Chagas disease patients and non-infected subjects (NI, 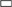 = 11). The profile of CD16, CD32, and CD64 was characterized at *ex vivo* **(A)** and upon *Trypanosoma cruzi*-antigen recall *in vitro* **(B)**. Data are displayed in box plot format (median with minimum and maximum values) as mean fluorescence intensity (MFI) for each Fc-γR. Significant differences at *p* < 0.05 are underscored by connecting lines and *p*-values provided in the figure.

The *ex vivo* analysis of Fcγ-R expression by phagocytes, NK-cells and B-cells from Chagas disease patients are presented in **Figure 1A**. Data demonstrated that IND displayed an overall higher expression of Fcγ-R (CD16, CD32, and CD64) by neutrophils and CD16 and CD64 by monocytes as compared to CARD. Conversely, CARD presented a down-regulation of CD16 expression by monocytes and an increased expression of CD32 by B-cells as compared to NI (**Figure 1A**).

### *In Vitro T. cruzi*-Antigen Recall Induces an Overall Decrease of Fc-γR Expression in Chagas Disease Patients Mainly in CARD

The results of Fcγ-R expression by peripheral blood leukocytes upon *T. cruzi*-antigen stimuli *in vitro* are shown in **Figure 1B**. The results were expressed as *T. cruzi*-antigen/CC index. Data analysis demonstrated a general decrease of Fc-γR expression by innate immunity cells (NEU, MON, and NK-cells) in Chagas disease patients with the major effect observed in CARD, except for CD16 expression by MON, which was up regulated in IND (**Figure 1B**).

### Preserved IL-10 Production Was the Hallmark of IND Whereas Lower Levels of IL-10^+^ Phagocytes, Along With Enhanced Frequency of TNF^+^ MON and NK-Cells, Were the Major Functional Features of CARD

**FIGURE 2:**
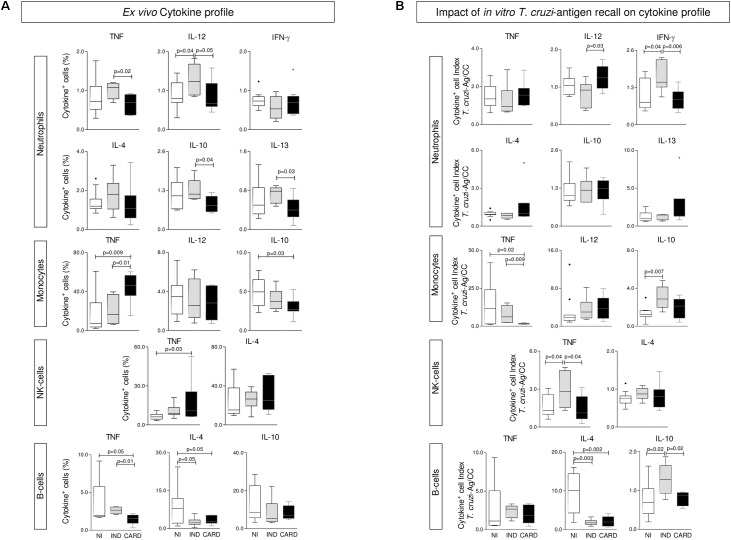
Cytokine profile of phagocytes, NK-cells, and B-lymphocytes. The analysis of cytokine pattern produced by neutrophils, monocytes, NK-cells, and B-cells were performed in whole blood samples collected from indeterminate (IND, 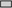 = 8) and cardiac (CARD, 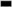 = 13) Chagas disease patients and non-infected subjects (NI, 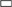 = 11). Pro-inflammatory/regulatory cytokines (TNF, IL-12, IFN-γ, IL-4, IL-10, and IL-13) were measured at *ex vivo* **(A)** and upon *T. cruzi*-antigen recall *in vitro* **(B)**. Data are displayed in box plot format (median with minimum and maximum values) as percentage of cytokine^+^ events for each cell population. Significant differences at *p* < 0.05 are underscored by connecting lines and *p*-values provided in the figure.

The *ex vivo* cytokine profile of phagocytes and NK and B cells from Chagas disease patients are presented in **Figure 2A**. The results showed that, regardless of the enhanced levels of IL-12^+^ NEU and lower IL-4^+^ B-cells, IND generally presented a baseline cytokine pattern in most circulating leukocytes and a preserved IL-10 production. Conversely, a classical TNF-mediated pro-inflammatory profile (MON and NK-cells) was observed in CARD, along with decreased frequency of IL-10^+^ phagocytes (**Figure 2A**).

### *In Vitro T. cruzi* Antigen-Recall Impacts the Functional Pattern of Leukocytes Mostly in IND, Leading to a Concomitant Increase of IFN-γ^+^NEU/TNF^+^NK-Cells and IL-10^+^MON/IL-10^+^B-Cells

The functional cytokine profile of peripheral blood leukocytes upon *T. cruzi*-antigen stimuli *in vitro* was assessed as *T. cruzi*-antigen/CC index and presented in **Figure 2B**. Data analysis pointed out that leukocytes from IND embraced the most change, as shown by the enhanced levels of IFN-γ^+^NEU and TNF^+^NK-cells, counterbalanced by an increased frequency of IL-10^+^MON and IL-10^+^B-cells (**Figure 2B**).

### Biomarker Signatures Further Emphasized the Contrasting Fc-γR Expression and Functional Cytokine Microenvironment Observed in IND and CARD

**FIGURE 3:**
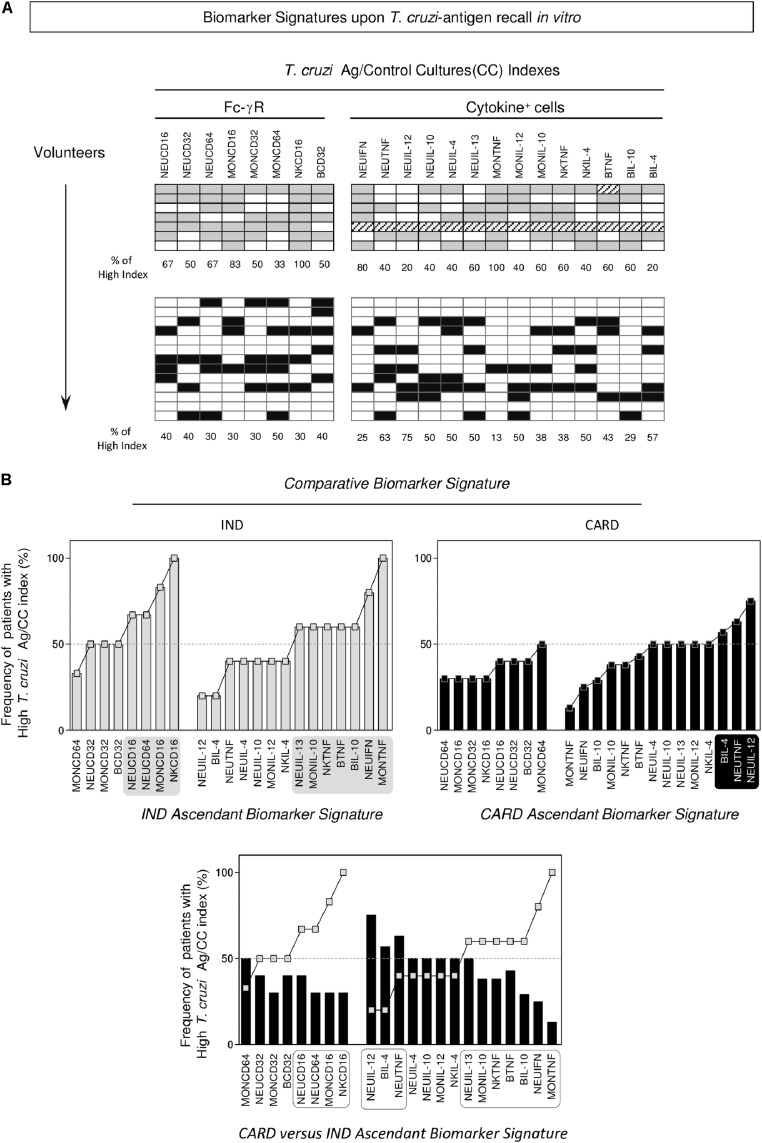
Biomarker signatures upon *T. cruzi*-antigen recall *in vitro.* **(A)** Gray-shaded diagrams were constructed to compile the proportion of volunteers presenting (*T. cruzi*-antigen/Control Culture) index above the global median cut-off established for the study population. Twenty-two attributes (Fc-γR and Cytokine^+^ cells) were assessed and the percentage of volunteers with a high index determined for indeterminate (IND, 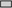 = 7) and cardiac (CARD, 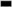 = 13) Chagas disease patients. **(B)** The biomarker signatures were assembled as an ascendant curve for each clinical group to identify the relevant attributes as those with frequency above the 50^th^ percentile (gray-shaded background). The biomarker signature of CARD was further overlaid with the IND-ascendant signature for comparative analysis. Differences were considered for attributes with frequencies confined into distinct 50^th^ percentiles (dashed-rectangles).

Data mining approaches were applied to build the biomarker signature, resulting in the phenotypic features of Fc-γR expression and functional profile (intracytoplasmic cytokine) of circulating leukocytes from IND and CARD (**Figure 3**, top panels). The frequency of patients with a “high” *T. cruzi*-antigen/CC index was calculated for each biomarker as a column statistic proportion and used to assemble the ascendant biomarker signatures of IND and CARD (**Figure 3**, middle panels). The results showed that IND presented up-regulated Fc-γR expression, particularly CD16, on NEU, MON and NK-cells, contrasting with a generally decreased Fc-γR expression observed in CARD (**Figure 3**, middle panels). Moreover, while IND presented a mixed functional pattern characterized by increased indices of TNF^+^ and IL-10^+^cells, CARD showed enhanced indices of TNF^+^ and IL-12^+^NEU (**Figure 3**, middle panels). Comparative analysis of the CARD biomarker signature (black bars) overlaid by the ascendant IND profile (gray line) underscored the gaps in Fc-γR expression in circulating leukocytes from CARD, mainly CD16 in NEU, MON, and NK (**Figure 3**, bottom panel). An analysis of functional features revealed relevant gaps in the cytokines microenvironment in CARD, specially TNF and IL-10. Conversely, up-regulation of TNF and IL-12 was noticed in NEU from CARD (**Figure 3**, bottom panel).

### Analysis of Anti-live *T. cruzi* Trypomastigote IgG Revealed That CARD Presented a Lower Seropositivity Ratio Along the Titration Curve as Compared to IND

**FIGURE 4:**
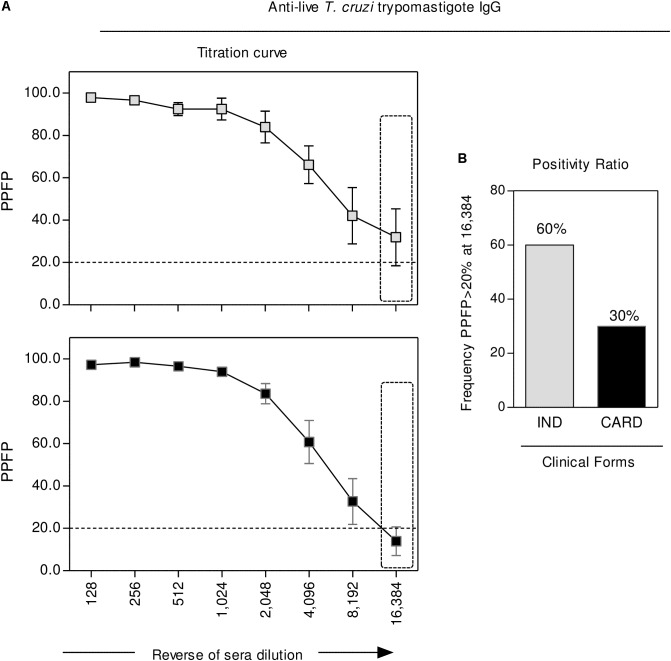
Anti-live *T. cruzi* trypomastigote IgG reactivity. The levels of anti-live trypomastigote antibodies were measured in serum samples collected from indeterminate (IND, 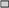 = 5) and cardiac (CARD, 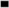 = 10) Chagas disease patients. **(A)** Data are presented as mean ± standard error value of Percentage of Positive Fluorescent Parasite (PPFP), along the titration curve (1:128 to 1:16,384). **(B)** The differential reactivity pattern observed at 1:16,384 serum dilution (dashed rectangle) was further addressed to identify the proportion of subjects with PPFP >20%. Data are reported as a positivity ratio for each clinical group.

The reactivity of anti-live *T. cruzi* trypomastigote IgG was carried out by the flow cytometric serological approach, with the results presented in **Figure 4**. Data are reported as the percentage of positive fluorescent parasites (PPFP) along the titration curve (128 to 16,384). Statistical analysis, using the PPFP = 20% as the threshold, demonstrated that, regardless the overlapped mean reactivity observed at low sera dilution, specifically at dilution 16,384, CARD presented a lower seropositivity ratio as compared to IND (**Figure 4**).

### Integrative Biomarker Network Analysis Revealed Distinct Assemblage of Fc-γR Expression, Cytokine Microenvironment, and Anti-*T. cruzi* IgG Interface in IND and CARD

**FIGURE 5:**
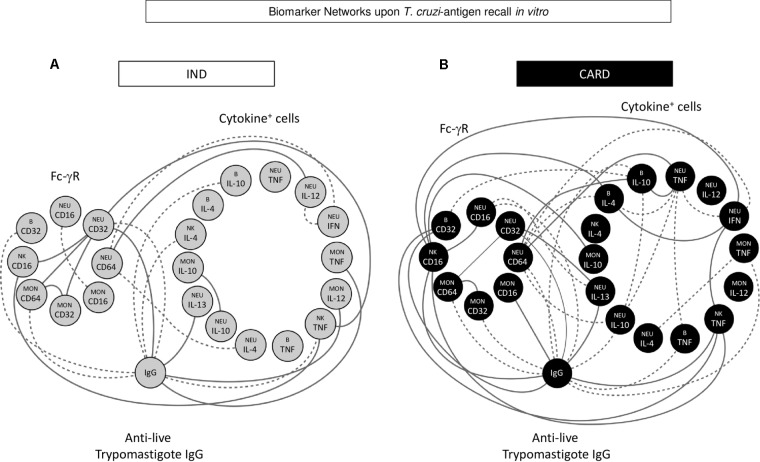
Biomarker networks upon *T. cruzi-*antigen recall *in vitro.* Nodular network layouts were assembled to characterize the association between Fc-γR expression and cytokine profile of peripheral blood leucocytes upon *T. cruzi* antigen recall *in vitro* and the anti-live trypomastigote antibody reactivity from IND **(A)** and CARD **(B)** Chagas disease patients. Clustered distribution of Fc-γR expression and cytokine microenvironment interfaced by the anti-live *T. cruzi* trypomastigote IgG reactivity provided an integrative overview of biomarker networks. Neighborhood connections identify the correlation scores between attributes, classified as positive (solid line) or negative (equal dashed line).

Machine learning methods were applied to identify significant connectivity among biomarkers. This approach provided insights into the cross-talk dynamic between Fc-γR expression and functional features of circulating leukocytes, resulting in the anti- *T. cruzi* antibody interface. **Figure 5** shows circular layouts representing three clusters of biomarkers (Fc-γR expression, cytokine microenvironment, and anti-live *T. cruzi* trypomastigote IgG interface) created to highlight the major differences between IND and CARD. Despite the similar proportion (50%) of positive and negative correlations observed in both clinical groups, CARD presented a more robust network with greater internode connectivity. Moreover, relevant intra-cluster differences were found, as shown by stronger Fc-γR positive inter-connections in IND and cytokine microenvironment negative inter-connections in CARD (**Figure 5**).

## Discussion

There have been numerous studies investigating the immunological mechanisms associated with distinct clinical outcomes of chronic Chagas disease. However, the complex and multifactorial immunological events involved in this phenomenon have not been completely elucidated. Additional investigation is still needed to characterize the involvement of distinct cell subsets and molecules implicated in the host/parasite interaction. An analysis of this dynamic process is essential for a better understanding of disease outcome and may contribute to developing strategies for patient management. In this study, we have employed systems biology strategies to investigate the relationship between Fc-γR expression by distinct cell subsets and the cytokine microenvironment interfaced with anti-*T. cruzi* IgG reactivity to provide novel insights into the development/maintenance of distinct clinical status of cardiac Chagas disease.

It is well-known that human phagocytes express low, median, and high affinity Fc-γR and previous studies have demonstrated that these receptors mediate effector functions, including antibody-dependent cell-mediated cytotoxicity (Dyall et al., 1999) and phagocytosis (Van De Wimkel and Capel, 1993). Our data has shown that, in the absence of any exogenous *in vitro* interference, IND displayed an overall higher expression of Fcγ-R (CD16, CD32, and CD64) by neutrophils and CD16 and CD64 by monocytes. Conversely, in the previous study, which was carried out under unstimulated/stimulated *in vitro* cultures in the presence of supplementary addition of autologous serum, the IND group presented lower levels of CD16, CD32, and CD64 expression by monocytes (Gomes et al., 2012). Moreover, the authors showed that the downregulation of Fc-γR expression by monocytes was even higher in IND in the presence of live trypomastigote organisms. It is possible that these differences are due to a turnover Fc-γR expression observed *in vitro* or differential impact of stimuli with live parasites or soluble antigens. In this context, the soluble antigens may favor the generation of immune complex anti-*T. cruzi* antibodies in the culture’s microenvironment that would ultimately interfere at the Fc-γR/cell interface.

Additionally, we have observed that the CARD group presented an increased expression of CD32 by B-cells. Muta et al. (1994) previously suggested that the binding of immune complex to the CD32 receptor on B-cells generates a negative regulatory sign that is associated with the inhibition of the activation, proliferation, antibody secretion by B lymphocytes, which explains the lower levels of *T. cruzi* specific IgG in the plasma from CARD patients observed in this study. Indeed, the higher levels of *T. cruzi* specific IgG antibodies by IND corroborate Cordeiro et al. (2001), suggesting an increased antibody-dependent cell-mediated cytotoxicity activity by these patients, which could be associated with protection.

It is well-known that cytokines play a pivotal role in the progression of different clinical forms of Chagas disease. A fine balance between the pro-inflammatory/regulatory profile of the cytokine microenvironment is important to controlling pathogenesis in chronic cardiac Chagas disease. Our data has shown that, while IND patients display a concomitant increase of IFN-γ^+^NEU/TNF^+^NK-cells and IL-10^+^MON/IL-10^+^B-cells, lower levels of IL-10^+^ phagocytes together with the enhanced frequency of TNF^+^ MON and NK-cells was the major functional feature of CARD. Corroborating our data, other groups have observed that monocytes from the IND group displayed a higher production of IL-10 upon exposure to *T. cruzi* antigens, while monocytes from CARD preferentially secrete high levels of TNF-α (Takeda et al., 2003; Souza et al., 2007). Indeed, Gomes et al. (2012) observed that indeterminate patients displayed a higher ratio of monocytes expressing IL-10 over TNF-α and IL-12, suggesting that the production of regulatory cytokines is important to counterbalancing the pro-inflammatory immune response that may cause tissue damage.

Together, our findings revealed that relevant gaps in Fc-γR expression, along with impaired regulatory cytokine microenvironment in the interface of anti-*T. cruzi* IgG reactivity, prompted an imbricate and robust biomarker network in patients with cardiac Chagas disease. A larger number of internode connectivity with overall negative connectivity may account for the development/maintenance of the clinical status of CARD patients. These data reinforce that CARD may have insufficient control of an inflammatory response, as compared with IND patients, which contributes to the establishment of cardiac pathology.

## Conclusion

Our study demonstrated that patients with different clinical forms of chronic Chagas disease presented changes in the peripheral blood leucocytes profile on the total *T. cruzi* specific IgG levels, as well as in the cytokines environment pattern, supporting the hypothesis that immunomodulatory mechanisms are involved in the control of immune response in the indeterminate clinical form of Chagas disease. This study provides an integrative overview that is original and brings novel insights to these imbricate microenvironments interfacing the humoral and cellular immune response in Chagas disease.

## Author Contributions

JG, FA, AT-C, JD, SE-S, and OM-F contributed with the conception and design of the study. DV-A, RS-A, and AW organized the database. JG, FA, IV, and OM-F performed the statistical analysis. JG, FA, and IV wrote the first draft of the manuscript. PL, DV-A, and R-SA wrote sections of the manuscript. All authors contributed to manuscript revision, read and approved the submitted version.

## Conflict of Interest Statement

The authors declare that the research was conducted in the absence of any commercial or financial relationships that could be construed as a potential conflict of interest.

## References

[B1] Brodskyn C. I. da Silva A. M. Takehara H. A. Mota I. (1988). Characterization of antibody isotype responsible for immune clearance in mice infected with *Trypanosoma cruzi*. *Immunol. Lett* 18 255–258. 10.1016/0165-2478(88)90171 3141272

[B2] Cordeiro F. D. Martins-Filho O. A. Da Costa Rocha M. O. Adad S. J. Correa-Oliveira R. Romanha A. J. (2001). Anti-*Trypanosoma cruzi* immunoglobulin G1 can be a useful tool for diagnosis and prognosis of human Chagas disease. *Clin. Diagn. Lab. Immunol.* 8 112–118. 10.1128/CDLI.8.1.112-118.2001 11139203 PMC96018

[B3] Cunha-Neto E. Duranti M. Gruber A. Zingales B. De Messias I. Stolf N. (1995). Autoimmunity in Chagas disease cardiopathy: biological relevance of a cardiac myosin-specific epitope cross-reactive to an immunodominant *Trypanosoma cruzi* antigen. *Proc. Natl. Acad. Sci. U.S.A.* 92 3541–3545. 10.1073/pnas.92.8.3541 7536937 PMC42203

[B4] Dyall R. Vasovic L. V. Clynes R. A. Nikolic-Zugic J. (1999). Cellular requirements for the monoclonal antibody-mediated eradication of an established solid tumor. *Eur. J. Immunol.* 29 30–37. 10.1002/(SICI)1521-4141(199901)29:01<30::AID-IMMU30>3.0.CO;2-D 9933083

[B5] Gomes J. A. Bahia-Oliveira L. M. Rocha M. O. Martins-Filho O. A. Gazzinelli G. Correa-Oliveira R. (2003). Evidence that development of severe cardiomyopathy in human Chagas disease is due to a Th1-specific immune response. *Infect. Immun.* 71 1185–1193. 10.1128/IAI.71.3.1185-1193.2003 12595431 PMC148818

[B6] Gomes J. A. Campi-Azevedo A. C. Teixeira-Carvalho A. Silveira-Lemos D. Vitelli-Avelar D. Sathler-Avelar R. (2012). Impaired phagocytic capacity driven by downregulation of major phagocytosis-related cell surface molecules elicits an overall modulatory cytokine profile in neutrophils and monocytes from the indeterminate clinical form of Chagas disease. *Immunobiology* 217 1005–1016. 10.1016/j.imbio.2012.01.014 22387073

[B7] Heath A. W. Martins M. S. Hudson L. (1990). Monoclonal antibodies mediating viable immunofluorescence and protection against *Trypanosoma cruzi* infection. *Trop. Med. Parasitol.* 41 425–428. 10.1128/IAI.73.12.7960-7966.2005 2127473

[B8] Kierszenbaum F. (1980). Protection of congenitally athymic mice against *Trypanosoma cruzi* infection by passive antibody transfer. *J. Parasitol.* 66 673–675. 10.1016/0035-9203(88)90272-6 6775071

[B9] Kumar S. Tarleton R. L. (1998). The relative contribution of antibody production and CD8^+^T cell function to immune control of *Trypanosoma cruzi*. *Parasite Immunol.* 20 207–216. 10.1046/j.1365-3024.1998.00154.x 9651921

[B10] Lu B. Alroy J. Luquetti A. O. PereiraPerrin M. (2008). Human autoantibodies specific for neurotrophin receptors TrkA, TrkB, and TrkC protect against lethal *Trypanosoma cruzi* infection in mice. *Am. J. Pathol.* 173 1406–1414. 10.2353/ajpath.2008.080514 18832578 PMC2570131

[B11] Luiza-Silva M. Campi-Azevedo A. C. Batista M. A. Martins M. A. Avelar R. S. da Silveira Lemos D. (2011). Cytokine signatures of innate and adaptive immunity in 17DD yellow fever vaccinated children and its association with the level of neutralizing antibody. *J. Infect. Dis.* 204 873–883. 10.1093/infdis/jir439 21849284

[B12] Minoprio P. Itohara S. Heusser C. Tonegawa S. Coutinho A. (1989). Immunobiology of murine *Trypanosoma cruzi* infection: the predominance of parasite-nonspecific responses and the activation of TCRI T cells. *Immunol. Rev.* 112 183–207. 10.1111/j.1600-065X.1989.tb00558.x 2514135

[B13] Muta T. Kurosaki T. Misulovin Z. Sanchez M. Nussenzweig M. C. Ravetch J. V. (1994). A 13-amino- acid motif in the cytoplasmic domain of Fc gamma RIIB modulates B-cell receptor signaling. *Nature* 369:340. 10.1038/368070a0 8183374

[B14] Pricop L. Salmon J. E. Edberg J. C. Beavis A. J. (1997). Flow cytometric quantitation of attachment and phagocytosis in phenotypically-defined subpopulations of cells using PKH26-labeled Fc gamma R-specific probes. *J. Immunol. Methods* 205 55–65. 10.1016/S0022-1759(97)00053-7 9236915

[B15] Qiu W. Q. de Bruin D. Brownstein B. H. Pearse R. Ravetch J. V. (1990). Organization of the human and mouse low-affinity Fc gamma R genes: duplication and recombination. *Science* 248 732–735. 10.1126/science.2139735 2139735

[B16] Ravetch J. V. Kinet J. P. (1991). Fc receptors. *Annu. Rev. Immunol.* 9 457–492. 10.1146/annurev.iy.09.040191.002325 1910686

[B17] Ravetch J. V. Lanier L. L. (2000). Immune inhibitory receptors. *Science* 290 84–89. 10.1126/science.290.5489.84 11021804

[B18] Santos-Lima E. C. Vasconcellos R. Reina-San- Martin B. Fesel C. Cordeiro-Da-Silva A. Berneman A. (2001). Significant association between the skewed natural antibody repertoire of Xid mice and resistance to *Trypanosoma cruzi* infection. *Eur. J. Immunol.* 31 634–645. 10.1002/1521-4141(200102)31:2<634::AID-IMMU634>3.0.CO;2-H 11180129

[B19] Souza P. E. Rocha M. O. Menezes C. A. Coelho J. S. Chaves A. C. Gollob K. J. (2007). *Trypanosoma cruzi* infection induces differential modulation of costimulatory molecules and cytokines by monocytes and T cells from patients with indeterminate and cardiac Chagas’ disease. *Infect. Immun.* 75 1886–1894. 10.1128/IAI.01931-06 17283096 PMC1865727

[B20] Takeda K. Kaisho T. Akira S. (2003). Toll-like receptors. *Annu. Rev. Immunol.* 21 335–376. 10.1146/annurev.immunol.21.120601.141126 12524386

[B21] Van De Wimkel J. G. J. Capel J. A. (1993). Human IgG Fc receptor heterogeneity: molecular aspects and clinical implications. *Immunol. Today* 14 215–221. 10.1016/0167-5699(93)90166-I 8517920

[B22] Vitelli-Avelar D. M. Sathler-Avelar R. Teixeira-Carvalho A. Pinto Dias J. C. Gontijo E. D. Faria A. M. (2008). Strategy to assess the overall cytokine profile of circulating leukocytes and its association with distinct clinical forms of human Chagas disease. *Scand. J. Immunol.* 68 516–525. 10.1111/j.1365-3083.2008.02167.x 18803607

[B23] World Health Organization [WHO] (2017). *World Health Organization- Chagas Disease (American Trypanosomiasis) Fact Sheet NÉ*. Geneva: WHO, 340.

